# Supplementary figures and images for: Identification of a homology-independent linchpin domain controlling mouse and bank vole prion protein conversion
Source: PLoS Pathog. 2020 Sep 8;16(9):e1008875. doi: 10.1371/journal.ppat.1008875 (PMC7508373; doi:10.1371/journal.ppat.1008875)

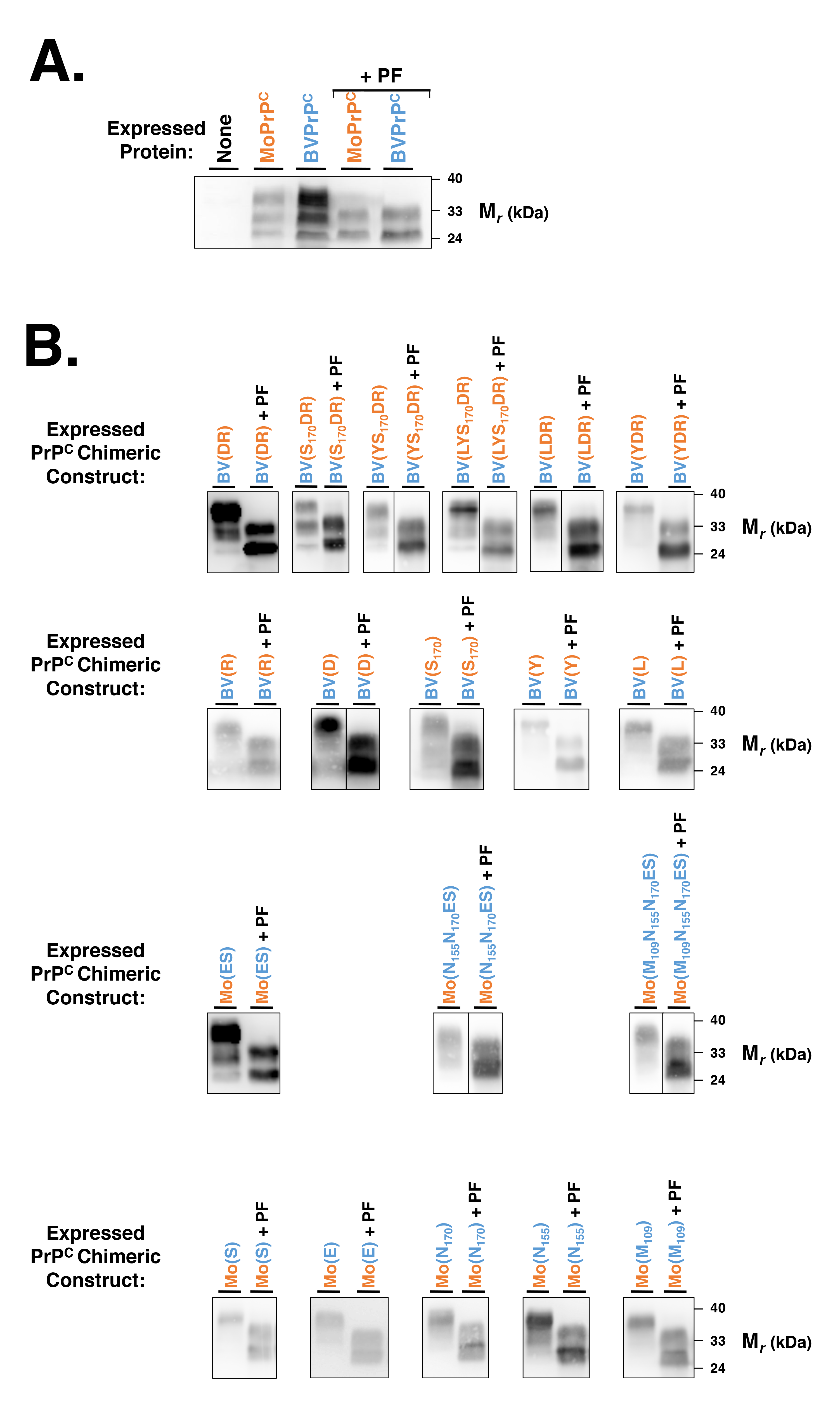

Supplement: S1 Fig — Western blots showing PrP partially purified from cell lysates expressing the indicated protein, or no protein, before and after partial enzymatic deglycosylation with PNGase F (+ PF), as indicated. (A) Wild-type constructs. (B) Chimeric constructs. (TIF) [file ppat.1008875.s001.tif]

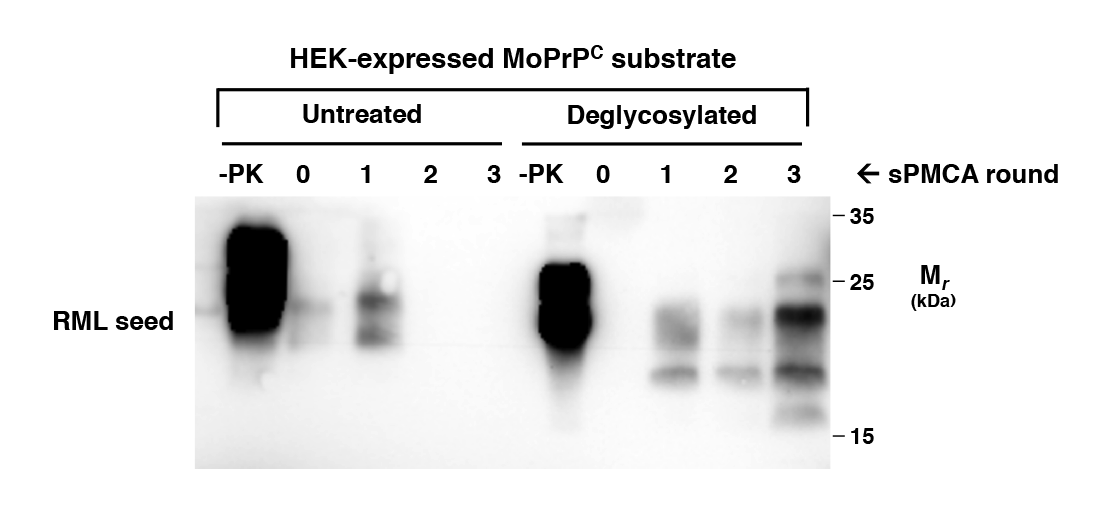

Supplement: S2 Fig — Western blots showing three-round reconstituted sPMCA reactions using partially purified Mo PrPC substrate supplemented with PrP0/0 BH and seeded with mouse RML. Substrates were either untreated or partially deglycosylated with PNGase F during purification, as indicated. -PK = samples not subjected to proteinase K digestion; all other samples were proteolyzed. (TIF) [file ppat.1008875.s002.tif]

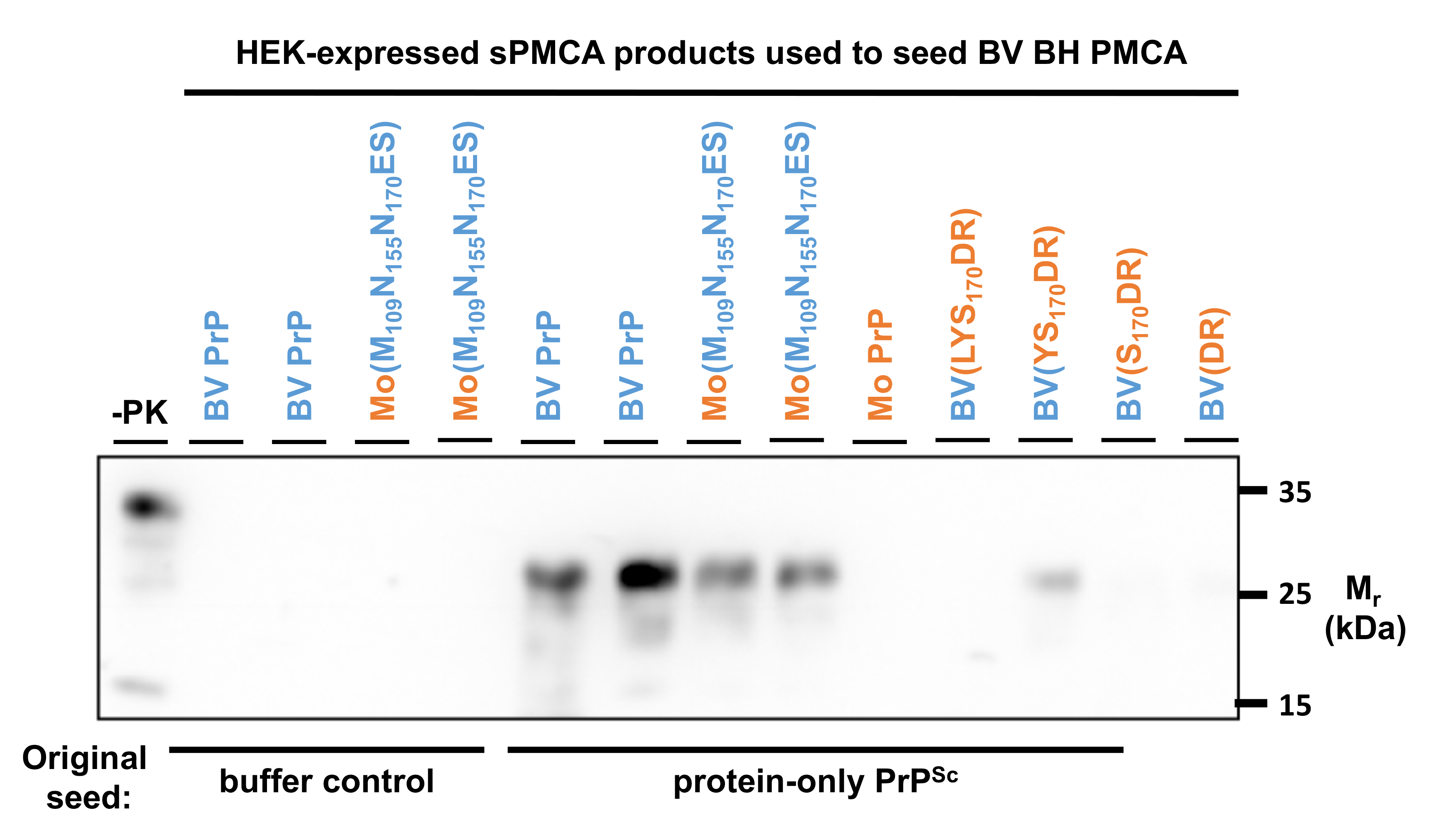

Supplement: S3 Fig — Western blots showing PMCA reactions using crude bank vole brain homogenate substrate seeded with various HEK-expressed PrPSc molecules, as indicated. The chimeric PrPSc molecules are final round products of 3-round sPMCA reactions using HEK-expressed PrPC substrates originally seeded with Mo protein-only recPrPSc or buffer, as indicated. (TIF) [file ppat.1008875.s003.tif]

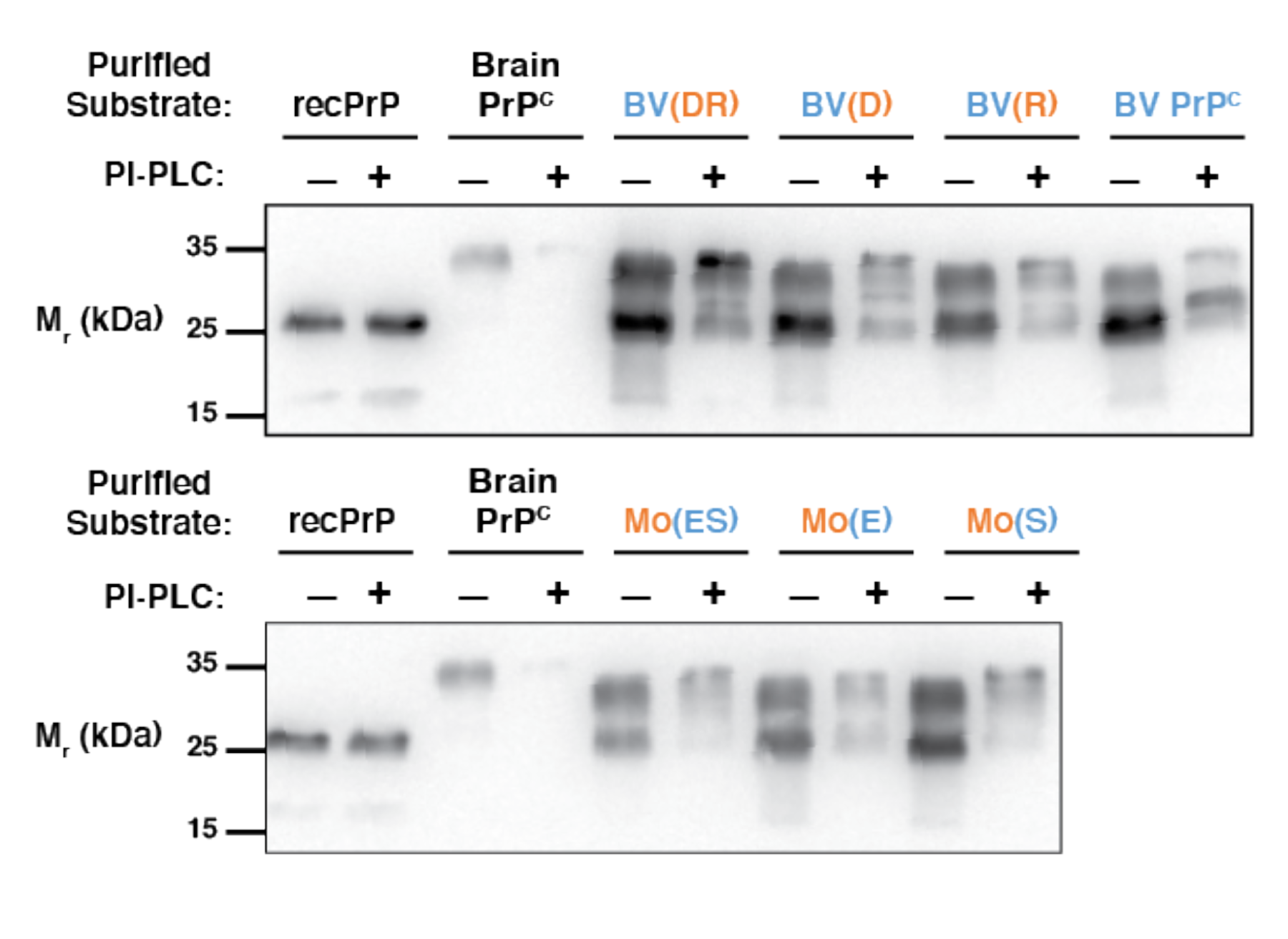

Supplement: S4 Fig — Western blots of various purified PrP substrates (bacterially-expressed recPrP, immunopurified native brain PrPC, different HEK-expressed PrPC chimeras with substitutions at residues 227 and/or 230 as indicated, or HEK expressed bank vole PrPC) treated either with (+) or without (-) 0.25U/mL Phosphoinositide phospholipase C (PI-PLC) for 14 hr at 37 oC with shaking at 800 r.p.m. as indicated. Proteins were transferred from a 12.5% polyacrylamide gel onto a Millipore Immobilon-P PVDF membrane by semi-dry electroblotting, and probed with mAb 27/33. (TIF) [file ppat.1008875.s004.tif]
