# Supplementary material for: Identification of a homology-independent linchpin domain controlling mouse and bank vole prion protein conversion
Source: PLoS Pathog. 2020 Sep 8;16(9):e1008875. doi: 10.1371/journal.ppat.1008875 (PMC7508373; doi:10.1371/journal.ppat.1008875)
Supplement: Attachment — Submitted filename: Point-by-point response v6.docx [file ppat.1008875.s005.docx]

Dear Drs.Gilch and Malim,

We thank both of you and all of the reviewers for the constructive critiques of our manuscript. We have added new data and revised the text to address all of the excellent suggestions, and agree that the revised version of the manuscript is much improved over the original. Please find a point-by-point response below, with our responses indicated by *green italicized font*.

Sincerely,

Surachai Supattapone

**Reviewer #1**

While the data and design are appropriate, it’s not clear that an artificially generated prion which does not convert its native substrate to PrPSc is a valid system to study the conversion mechanism of natural prions. The argument that the inability of recPrPSc to convert mouse PrPC enables the study of important conformational determinants without taking into consideration amino acid sequence does not make much logical sense. The primary sequence of a protein is intimately tied to conformation, and earlier studies have already made the point that conformational differences driven by amino acid sequence significantly impact PrPSc formation.

*We agree that protein-only PrP^Sc^ is not a natural prion, and did not intend to imply that assays using this non-infectious, synthetic conformer are sufficient to study the conversion mechanism of natural prions. Instead, we only intended to use it as a screening tool to help identify residues that impact the conformational change of native bank vole PrP^C^ into PrP^Sc^*. *In this capacity, it helps us avoid the confounding factor of sequence similarity between seed and substrate during the initial screen to systematically and rapidly identify candidate linchpin residues, but we completely agree that additional studies both in vitro (like the one shown in Fig. 7) and in vitro are required to characterize the role played by the residues identified in our screen for the propagation of specific natural prion strains.* *We have modified the discussion section to clarify this important point.*

1) All of the Mo-BV chimeric constructs are expressed in HEK 293 cells. However, no data are shown to indicate whether or not the various chimeras are processed normally, i.e. fully glycosylated, GPI anchored, and expressed on the cell surface. Aberrant processing of the chimeric PrPC molecules, especially those with mutations at 227 and 230 in the C-terminus where the GPI anchor is attached, could affect their ability to convert to PrPSc. The authors should provide information on whether or not the chimeras tested were appropriately processed by the cell.

*Because all of our constructs are chimeras of WT mouse and bank vole PrP^C^, we did not anticipate any problems with post-translational processing. Nonetheless, we certainly agree that it is important to consider and check for the possibility that some of the substitutions may have altered glycosylation, GPI-anchoring, proper folding, or the general ability to undergo convert into PrP^Sc^.*

a. *Glycosylation: We have added new panels to Supporting Information S1 Fig showing the effects of PNGase F treatment for all of the chimeric proteins used in the manuscript. This additional data confirms that all of the HEK-expressed chimeras are properly glycosylated.*
b. *GPI-anchor: We have also added a new Supporting Information S4 Fig which uses PI-PLC digestion to show that the 6 chimeras that contain either single or double substitutions near the C-terminal attachment site (i.e. residues 227 and 230) are all properly anchored.*
c. *Proper folding: It is likely that all of the chimeric substrates used in our experiments are properly folded because they remain in the supernatant after centrifugation at 100,000 x g for 1 hr and can be bound to and eluted from both affinity and ion exchange columns during the solubilization and purification processes described in Methods. We have modified the Methods section to address this important point.*
d. *Ability to convert into PrP^Sc^: Nearly all of the chimeric substrates successfully propagated the positive control mouse prion seed RML in sPMCA. The one exception was Mo(N_170_ES), which was unable to propagate RML PrP^Sc^ for unknown reasons. Despite its ability to propagate RML PrP^Sc^, this substrate was still able to propagate Mo protein-only recPrP^Sc^. Nonetheless, we removed this particular chimera from our data set to avoid any possibility of misinterpretation.*

2) Based on the data in Fig. 3B, the author states that the BV(DR) substrate with two amino acid changes converts less well than the BV(LDR) and BV(YDR) substrates with 3 amino acid substitutions (compare row 2 to rows 6 and 7). However, this is not clear from the data shown in Figure 3 since the signal intensities on the gels shown are very similar. If possible, the authors should include quantitation data to show this more convincingly.

*We have added quantitation by replacing check and cross symbols within the A panels for each figure with % conversion efficiency values determined by densitometry. The quantitation data do confirm that BV(LDR) and BV(YDR) convert more efficiently than BV(DR). The BV(DR) construct is notably variable; we have added a comment to note this point in the results section.*

3) In Figure 7, recPrPSc does not convert the BV(DR) construct to PrPSc. However, in Figure 3 it does convert BV(DR), albeit at a lower efficiency. This suggests some variability in conversion efficiency between experiments that the authors should address.

*We agree with this observation. We and others do observe a degree of stochastic behavior in sPMCA experiments. In addition, the variability in conversion efficiency observed with this chimera in particular may also reflect that it is on the cusp of being a viable substrate, i.e. it has a marginal ability to convert. This explanation is supported by the observation that additional substitution of S170 completely knocks out conversion, as shown in Fig 3A. We have modified the text to discuss the observed variability.*

4) In Figure 7, conversion of mouse (ES) by hamster, sheep, and deer PrPSc brain homogenate yields conversion products in the first 2 rounds that are protease-resistant but do not appear to decrease in size. By the third round, there does appear to be some protease-resistant, truncated product in the sheep and deer reactions. Do the authors have any explanation for why Mo(ES) appears to be fully protease resistant through two rounds of sPMCA only to become partially protease-resistant by the third round?

*The observed changes observed in protease-resistant PrP^Sc^ migration may be caused by conformational/strain adaptation, a process that can occur very rapidly during sPMCA(Meyerett et al., 2008). We have modified the text to add this possible explanation.*

5) A mouse PrPC substrate where the two glycosylation sites have been mutated yielding an unglycosylated product (G3) is not converted to PrPSc by recPrPSc. How does this result impact interpretation of the other experiments in the paper, all of which use partially purified, PNGaseF treated, deglycosylated PrPC substrate? If fully glycosylated PrPC substrate is used, how does that effect the ability of protein only recPrPSc to convert the substrate to PrPSc?

*Unfortunately, fully glycosylated HEK-expressed PrP^C^ is not a good substrate for mouse prion seeds, including RML, due to hyper-glycosylation in HEK cells. We have added a new Supporting Information S2 Fig to show this point. This is likely due to the previously observed inhibitory effects of PrP^C^ glycosylation on the propagation of mouse prion strains(Burke et al., 2020; Nishina et al., 2006), which may be due to negatively charged sialic acid groups within the glycan structure(Katorcha et al., 2015).*

The figure legend to Figure 7 describes panel C but this panel is not in the figure shown. This should be corrected.

*We apologize for this oversight, and have corrected it in the revised manuscript.*

**Reviewer #2**

Prion conversion is a very complex process and many factors contribute to it, and the main influencer is PrP sequence. The current study identifies E227 and S230 as the key amino acids that make Bank Vole PrPC the almost universal substrate for prion conversion by a variety of prion strains, including the in vitro formed protein-only recombinant PrPSc. Although the substitutions of D227 and R230 of mouse PrPC to E227 and S230, respectively, render the mouse chimeric PrPC susceptibility to various prion strains, which strongly supports the key role of E227 and S230, it requires more evidence to conclude such domain as the “general” linchpin for prion conversion, since the only two PrPC species investigated are Bank Vole and mouse. It would be much more convincing if hamster PrPC with E227 and D230 mutations could also be converted by RML, Scrapie and CWD.

*We think that this is an excellent experimental suggestion, and we would very much like to do it. However, we are unable to do so in a timely fashion because we do not have these constructs in hand, and the core facility that helps us express proteins in large-scale suspension culture (using a large orbital shaker inside a CO_2_ incubator) is indefinitely closed due to COVID-19.*

*In general, we agree with the reviewer that we have only tested PrP^C^ sequences from two species and a few prion strains in our study, and therefore much more evidence (both in vitro and in vivo) is required to conclude that E227 and S230 is a general linchpin domain, and have modified the title, abstract, and conclusion to be more specific for mouse and bank vole PrP.*

1. One of the limitations of this study, as the authors have pointed in the discussion part, is the lack of a way to confirm the infectivity of various chimeric PrPSc produced in the PMCA reactions. The authors argue that “However, we have previously shown that similar reactions containing BV PrPC substrate and seeded with protein-only recPrPSc produces fully infectious prions, making it likely that we are also studying a process relevant to infectious prion formation in this study”. While it is impractical to ask for in vivo bioassays, the author may consider evaluating the seeding ability of various chimeric PrPSc by using BV and mouse brain homogenates containing WT PrPC as substrates in PMCA reactions, which could serve as the prion infectivity surrogate.

*We have added a new Supporting Information S3 Fig to show that chimeric sPMCA products can subsequently seed brain homogenate PMCA reactions.*

2. In figure 1, the numbering for all PrP sequences follows the hamster PrPC sequence (23-231). However, in figure 8, the numbering follows mouse PrP sequence (23-230).

*We have changed the numbering in revised Figure 8 so that it also follows hamster numbering.*

3. In figure 5B, Mo(N155N150ES) on the third row should be Mo(N155N170ES).

*We apologize for this oversight, and have corrected the figure labeling in the revised manuscript.*

**Reviewer #3**

1. Because all the study is based on the difference of PrPSc levels in the PMCA products over serial rounds, the manuscript should include robust quantification and statistical analysis of PrPSc when mutation effects are compared.
This is especially important as i) there is no indication on the reproducibility of the experiments shown, ii) PrPSc abundance is generally low in the western blots (compare for instance Fig3 panel B, 2nd vs 3rd lane to measure the effect of the S170 substitution, a key residue of the discussion), iii) PrPres electrophoretic pattern and contribution of initial PrPSc seed to the PrPSc signal detected in the first round are difficult to estimate.

*Thank you for the suggestion. We have added quantitation by replacing check and cross symbols within the A panels for each figure with % conversion efficiency values determined by densitometry ± standard error values.*

2. The PrPres electrophoretic patterns are difficult to identify. The pattern is frequently polluted by bands of low Mr. Sometimes PK digestion are insufficient, resulting in PrPres patterns migrating like PrPC (see Fig 7 last lane for instance). The pattern is not consistent among the experiments, compare for instance RML seed with Bank vole PrPC fin Fig7B first lane and Fig 2A first lane.

*We agree with these astute observations. In some cases, the pattern inconsistency (both in between rounds of one experiment and between different experiments) arise from the stochastic nature of PrP^Sc^ formation during sPMCA, including the possibility of rapid strain adaptation(Meyerett et al., 2008). This is more likely to occur during cross-species propagation(Castilla et al., 2008) such as when mouse RML is used to seed BV PrP^C^. We have modified the text to discuss this point.*

3. The authors expressed chimeric PrPC in human HEK293 cells and purified PrPC before using it as substrate in PMCA experiments:
3.1 The authors do not mention whether these cells express or not human PrPC. Human PrPC could be converted or interfere with chimeric PrP in PMCA reactions.
3.2 Chimeric PrPC is partly deglycosylated with PNGase F (which usually works on denatured material containing SDS?) before use as substrate. Partial deglycosylation may impact the relative convertibility of the chimeric species. In favor of this hypothesis, the authors show that removing one glycan on mouse PrPC facilitates conversion by Mo-recPrPSc, without any other amino acid mutation.
3.3 HEK cells are likely to express variable amounts of PrPC depending on the mutation performed. There is no indication on the concentration of PrPC used in the PMCA reaction. This is a key point as other authors showed that efficient conversion of cell PrPC substrate in PMCA requires concentrated material (PMID: 27384922). In other words, conversion may fail because of insufficient PrP levels, not mutation.

*We do not detect human PrP^C^ in untransfected HEK293 cells by western blot (Supporting Information S1 Fig, Expressed protein: None). Partial deglycosylation is necessary because HEK293 cells hyper-glycosylate PrP^C^, limiting its usefulness as a substrate for mouse prions. We have added a new Supporting Information S2 Fig to show this point. This is likely due to the previously observed inhibitory effects of PrP^C^ glycosylation on the propagation of mouse prion strains(Burke et al., 2020; Nishina et al., 2006), which may be due to negatively charged sialic acid groups within the glycan structure(Katorcha et al., 2015). We have inserted additional panels to Supporting Information S1 Fig to show that all of the chimeric substrates appear to be equally deglycosylated. We have also modified the text to consider the possibility that partial deglycosylation might impact the relative conversion efficiencies of different chimeras.*

*HEK293-expressed PrP^C^ substrates are used in sPMCA reactions at a final concentration of 4-5 µg/mL, based on quantitation of western blot signals with reference to a known quantity of immunopurified PrP^C^. We have added this information to the Methods section of the revised text. In all experiments, we perform an internal positive control for each chimeric substrate is processed in parallel to confirm that the PrP^C^ quality and concentration are adequate for serial propagation of RML.*

4. The added value of performing experiments on PrPC glycosylation mutants and on ΔGPI PrPC from transgenics is uncertain. For me, it is a different story.
Regarding ΔGPI PrPC, another confounding issue is that these mice spontaneously accumulate PrPSc. This may interfere with the PMCA reaction. In addition, PrPC from these mice is lowly glycosylated and this may also impact the conversion by Mo-recPrPSc. Thus ,the relative contribution of the GPI anchor is highly uncertain.

*We have removed the experiments using ΔGPI PrP^C^ substrate from the revised manuscript.*

Regarding the second chapter of the discussion (starting with “It is worth noting”) stating that residues E227 and S230 might stabilize PrPC, making it less prone to conversion (do the authors mean self-conversion or templated conversion?). How do the authors reconciliate this idea with the observation that I109 polymorphism is sufficient for spontaneous bank vole PrPC conversion into prions?

*To clarify, we hypothesize that E227 and S230 might destabilize PrP^C^. Our work suggests that these C-terminal residues play a role in templated conversion, but the work previously reported by Kobayashi et al.(Kobayashi et al., 2019) suggests that they also play a role in spontaneous conversion. Spontaneous formation of BV I109 prions also occurs in the context of E227 and S230, so it is possible that the I109 polymorphism promotes PrP conformational change through an independent destabilizing effect, but it may require E227 and S230 to do so. It would be interesting to see whether D227 and R230 substitutions might be able to prevent the spontaneous disease in mice over-expressing I109 BV PrP.*

Please add page and lanes number to facilitate the comments.

*We apologize for the omission, and have added page and line numbers to the revised document.*

The introduction is conveying incomplete information, particularly for a general audience:
As examples:
- Introduction, 2nd chapter, about the transmission barriers. “Humans are susceptible to CJD…”. This is obvious and not an example of a cross-species transmission. 2nd sentence: “…but not to CWD or scrapie”. The references mentioned here refer to transgenic modelling to study whether human PrP can be converted by CWD or scrapie. Whether humans are in fine susceptible or not, we do not know. They are counter-examples of conversion, see for instance the work published by Cassard et al. (PMID: 25510416 ), same for CWD with studies in primates.
- Resuming the species to a paper from 1999 by S. Priola is a big shortcut. The topics would deserve more than that. Transmission barriers are more than “dictated by amino acid sequence complementarity between the incoming PrPSc seed and PrPC substrate”. There are remarkable examples showing that the strain type is a key determinant too. For example, propagation of human CJD in bank vole without species barrier, efficacy of atypical BSE to propagate in human PrP mice, while classical BSE does not, etc. The description of the transmission barrier is too simplistic.

*We thank the reviewer for the helpful feedback, with which we agree. Originally, we had not meant to oversimplify the topic of transmission barriers, but rather to highlight the contribution of seed/substrate sequence similarity since that is the particular confounding factor that our approach uniquely overcomes. We recognize that additional factors such as strain type also play a crucial role, and have modified this section of the Introduction section accordingly.*

**References cited**

*Burke, C.M., Walsh, D.J., Mark, K.M.K., Deleault, N.R., Nishina, K.A., Agrimi, U., Di Bari, M.A., and Supattapone, S. (2020). Cofactor and glycosylation preferences for in vitro prion conversion are predominantly determined by strain conformation. PLoS Pathog 16, e1008495.*

*Castilla, J., Gonzalez-Romero, D., Saa, P., Morales, R., De Castro, J., and Soto, C. (2008). Crossing the species barrier by PrP(Sc) replication in vitro generates unique infectious prions. Cell 134, 757-768.*

*Katorcha, E., Makarava, N., Savtchenko, R., and Baskakov, I.V. (2015). Sialylation of the prion protein glycans controls prion replication rate and glycoform ratio. Scientific reports 5, 16912.*

*Kobayashi, A., Matsuura, Y., Takeuchi, A., Yamada, M., Miyoshi, I., Mohri, S., and Kitamoto, T. (2019). A domain responsible for spontaneous conversion of bank vole prion protein. Brain Pathol 29, 155-163.*

*Meyerett, C., Michel, B., Pulford, B., Spraker, T.R., Nichols, T.A., Johnson, T., Kurt, T., Hoover, E.A., Telling, G.C., and Zabel, M.D. (2008). In vitro strain adaptation of CWD prions by serial protein misfolding cyclic amplification. Virology 382, 267-276.*

*Nishina, K.A., Deleault, N.R., Mahal, S.P., Baskakov, I., Luhrs, T., Riek, R., and Supattapone, S. (2006). The stoichiometry of host PrPC glycoforms modulates the efficiency of PrPSc formation in vitro. Biochemistry 45, 14129-14139.*
