# Supplementary material for: Identification of a homology-independent linchpin domain controlling mouse and bank vole prion protein conversion
Source: PLoS Pathog. 2020 Sep 8;16(9):e1008875. doi: 10.1371/journal.ppat.1008875 (PMC7508373; doi:10.1371/journal.ppat.1008875)
Supplement: Attachment — Submitted filename: Response Letter 2.docx [file ppat.1008875.s006.docx]

August 1, 2020

Sabine Gilch, PhD
Pearls Editor
PLOS Pathogens

Michael Malim
Section Editor
PLOS Pathogens

Dear Drs. Gilch and Malim,

We thank you and the reviewers for helping to improve our manuscript. In response to Reviewer 1’s minor comment, we have removed references to Mo(N170ES) in both Table 1 and SFig 1. We do not wish to publish the peer review history.

Sincerely,

Surachai Supattapone
